# Identification and comparison of novel circular RNAs with associated co-expression and competing endogenous RNA networks in pulmonary tuberculosis

**DOI:** 10.18632/oncotarget.22710

**Published:** 2017-11-27

**Authors:** Xing Zhang, Min Zhu, Rong Yang, Weifeng Zhao, Xiaolong Hu, Jianhe Gan

**Affiliations:** ^1^ Department of Infectious Disease, First Affiliated Hospital of Soochow University, Suzhou, Jiangsu Province, 215006, China; ^2^ School of Biology and Basic Medical Science, Soochow University, Suzhou, Jiangsu Province, 215123, China

**Keywords:** pulmonary tuberculosis, circular RNAs, competing endogenous RNA, expression pattern, whole transcriptome sequencing

## Abstract

Pulmonary tuberculosis (PTB) is caused by *Mycobacterium tuberculosis* and is one of the most serious diseases worldwide. Circular RNAs (circRNAs) are a large class of non-coding RNAs that were identified with potential regulatory roles in disease pathogenesis and progression. In this study, we used whole transcriptome sequencing to identify circRNAs from 3 PTB patients and 3 healthy individuals to determine the expression pattern of circRNAs in blood and the circRNA molecular regulatory networks in PTB pathogenesis. One hundred and seventy differentially expressed (≥ 2-fold change) circRNAs were dysregulated in PTB, compared with in healthy individuals. Quantitative real-time polymerase chain reaction was used to validate the RNA sequencing analysis from 20 PTB patients, and the results were consistent with the sequencing data. Gene Ontology annotation and Kyoto Encyclopedia of Genes and Genomes pathway analysis were applied to explore the potential circRNA functions of the significantly deregulated genes. Several immunity pathways, including endocytosis pathways in cancer, mitogen-activated protein kinase signaling pathway, human T-lymphotropic virus type 1 infection, and ubiquitin-mediated proteolysis, were involved in PTB pathogenesis. Competing endogenous RNAs (ceRNA) were constructed and inferred that aberrant expression of circRNA-associated ceRNA resulted in extensive variation in gene expression by miRNA-mediated circRNA-mRNA crosstalk interactions. Our study revealed that the circRNA–miRNA–mRNA network may shed light on the biological functions of circRNAs in PTB and provide useful information for exploring potential roles of circRNA in PTB.

## INTRODUCTION

Pulmonary tuberculosis (PTB) is a chronic disease caused by *Mycobacterium tuberculosis* that presents a serious threat to human health worldwide. In 2015, 10.4 million people developed TB and 1.4 million died. Globally in 2015, an estimated 480,000 people developed multidrug-resistant TB. In China, PTB remains a serious public health threat [1]. Early diagnosis of PTB in clinical samples is important for control and treatment of the disease [2]. Traditional diagnostic methods for PTB, such as sputum smear microscopy, bacteriological detection and polymerase chain reaction (PCR), X-ray diagnosis and purified protein derivative test, cannot effectively diagnose PTB, because of low sensitivity and poor specificity. Delay in diagnosis and treatment of PTB has led to a large number of patients with drug-resistant PTB [3]. So, novel, rapid, sensitive and efficient biomarkers or methods are urgently needed to prevent the spread of PTB [4].

Numerous studies have revealed the importance of non-coding RNAs in the pathogenesis of PTB, supporting the importance of epigenomic regulation in disease progress [5, 6]. Circular RNAs (circRNAs) are a novel class of endogenous non-coding RNAs that play important roles in regulation of gene expression at a post-transcriptional level [7]. circRNAs have been demonstrated to have potential regulatory roles in pathogenesis and progression of many diseases [8]. Recent research has revealed aberrant expression of several circRNAs in many cancers and other diseases. Some circRNAs have the potential to act as diagnostic biomarkers, and other dysregulated circRNAs play important roles in cancer development. For example, hsa_circ_0013958 is reported to be a potential non-invasive biomarker for early detection and screening of lung adenocarcinoma [9]; hsa_circ_0000190 may be a novel non-invasive biomarker for the diagnosis of gastric cancer [10]; and hsa_circ_0004277 is a potential diagnostic biomarker for acute myeloid leukemia [11]. Examples of circRNAs involved in the development of cancer include the following. CCDC66 promotes colon cancer growth and metastasis, the expression of cANRIL with increased risk of atherosclerotic vascular disease [12]; cir-ITCH plays an important role in the development and progression of esophageal squamous cell carcinoma [13]; hsa_circ_0045714 regulates extracellular matrix synthesis as well as proliferation and apoptosis of chondrocytes by promoting expression of miR-193b target gene *IGF1R* [14]; hsa_circ_0010729 regulates vascular endothelial cell proliferation and apoptosis by targeting the miR-186/HIF-1α axis [15]; hsa_circ_0001564 acts as miR-29c-3p sponge to mediate tumorigenicity, which could act as a potential biomarker for osteosarcoma and provide a novel insight into competing endogenous (ce)RNA mechanisms in osteosarcoma [16]; and circ-0016347 acts as a positive regulator in osteosarcoma cell proliferation and invasion [17]. However, the expression profiles of circRNAs in PTB are not well characterized. Whether these circRNAs also contribute to the development of PTB or can be used as novel diagnostic biomarkers remains to be determined.

In this study, we investigated the expression patterns of circRNAs in PTB patients and healthy individuals and explored the regulatory mechanism of circRNAs in PTB. A total of 170 differentially expressed circRNAs and 2565 differentially expressed mRNAs were identified by comparing 3 PTB patients and 3 healthy individuals. In addition to the associated pathways and gene ontology items, we also performed ceRNA analysis for the first time in PTB. Our analysis revealed that several immunity pathways were involved in PTB pathogenesis, including endocytotic pathways in cancer, mitogen-activated protein kinase (MAPK) signaling pathway, human T-lymphotropic virus (HTLV)-1 infection, and ubiquitin-mediated proteolysis. We also found that dsyregulation expression of circRNA ceRNA resulted in deregulation of target gene expression by miRNA-mediated circRNA-associated ceRNA crosstalk interactions.

## RESULTS

### circRNA profile overview

**Figure 1:**
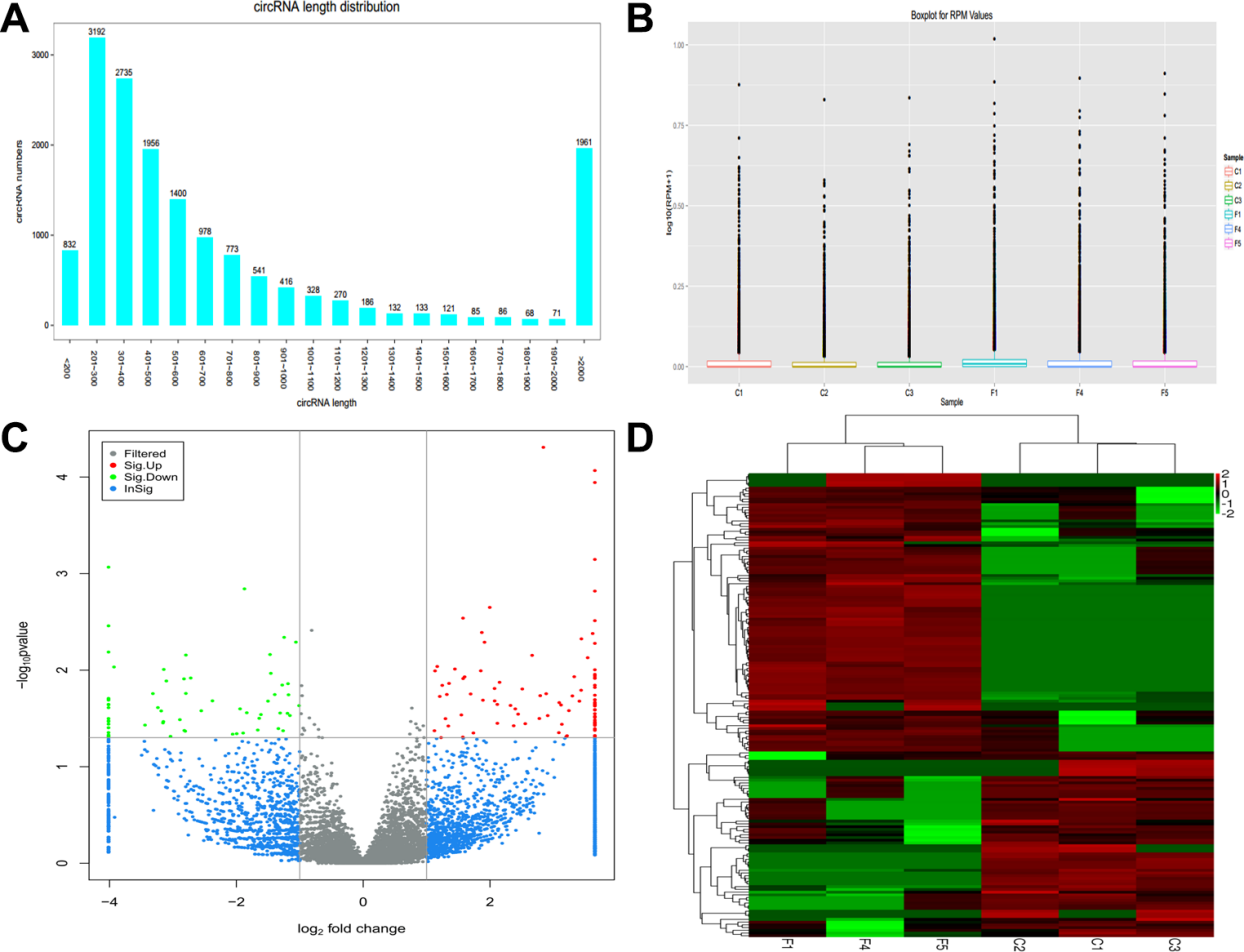
Expression pattern of circRNAs was detected by whole transcriptome sequencing in PTB patients and healthy individuals (**A**) Length distribution of circRNAs. (**B**) Box plots of RPM value of circRNAs in six groups. (**C**) Volcano plot shows the number and distribution of circRNAs in the same plane. (**D**) Heat map showing differentially expressed circRNAs from PTB patients compared with healthy individuals. Each row represents one circRNA, and each column represents each sample. Red indicates upregulation; green indicates downregulation. C1, C2 and C3 represent healthy individuals, and F1, F4 and F5 represent PTB patients.

Whole transcriptome sequencing was used to detect circRNA expression profile in 3 PTB patients and 3 healthy individuals using the Circbase database. We identified 16288 circRNAs from the PTB patients and healthy controls. The size of the circRNAs ranged from < 200 nt to > 2000 nt; most of which had a predicted spliced length of < 1000 nt, comprising 53.5% of circRNAs with length < 500 nt and 25.2% with length of 500–1000 nt (Figure 1A). Expression of circRNAs in PTB patients and healthy individuals was measured based on RPM (mapped back-splicing junction reads per million mapped reads), which indicated no abnormal expression in the six samples (Figure 1B). Significant differences in circRNAs between PTB patients and healthy individuals had a ≥ 2-fold change and *p* value < 0.05 and FDR < 0.05, as determined by DESeq method. The number and distribution of circRNAs in the same plane were displayed in the volcano plot, and had to satisfy both the above conditions (Figure 1C). Samples and circRNAs were separately classified and gene expression in different samples was assessed using a heatmap (Figure 1D).

**Figure 2:**
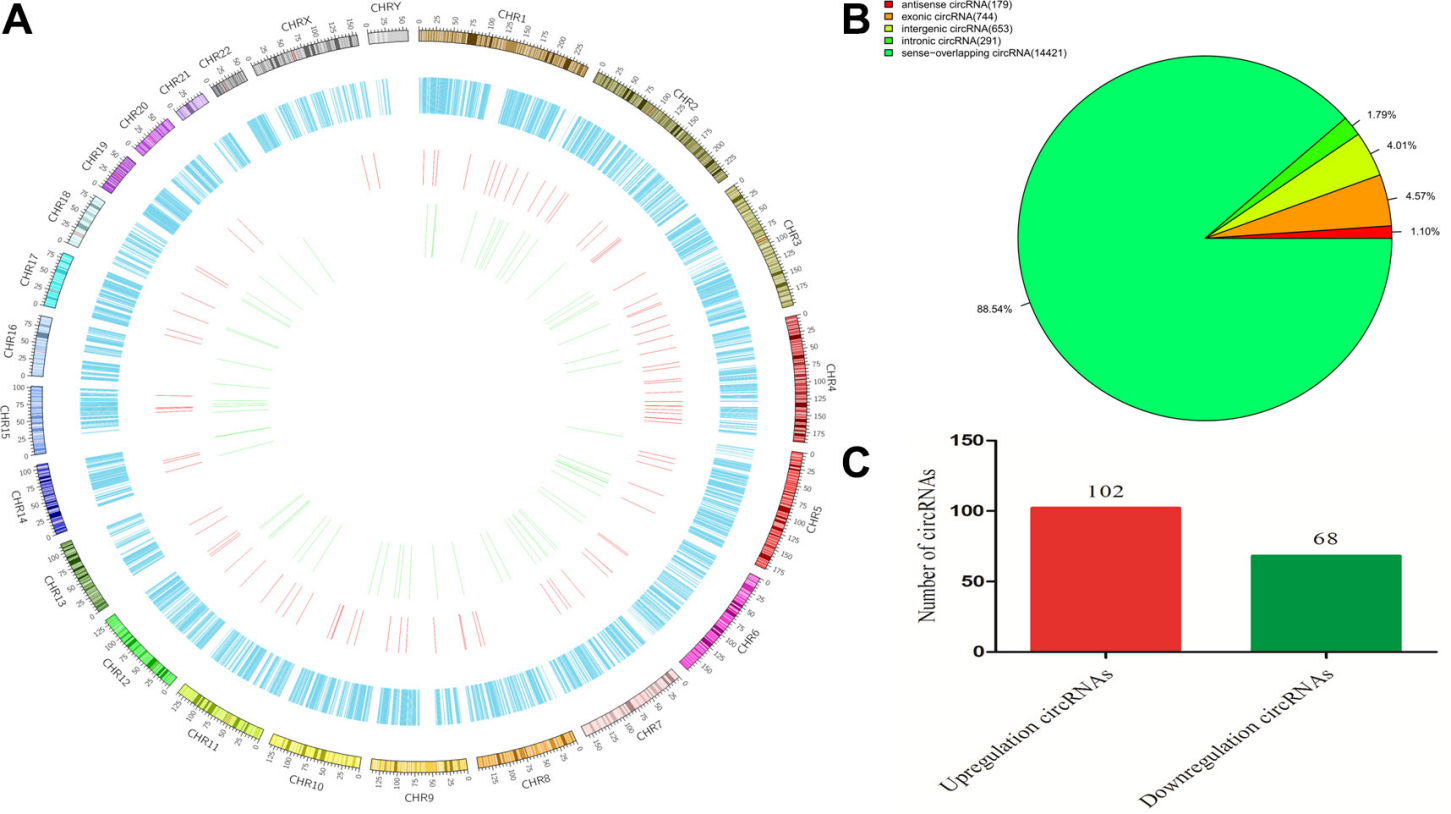
Identification of differentially expressed circRNAs from the PTB and healthy individuals (**A**) Circos plot showing circRNAs on human chromosomes. The outermost layer of the circos plot is a chromosome map of the human genome. The increased or decreased circRNAs have been marked in red or green bars, respectively. The second outermost circle represents all target circRNAs detected by sequencing, and the larger inner circle indicates the significantly by whole transriptome differentially upregulated circRNAs with fold change ≥ 2.0, *p* < 0.05 and FDR < 0.05. The smaller inner circle indicates the significantly differentially downregulated circRNAs with fold change ≥ 2.0, *p* < 0.05 and FDR < 0.05. (**B**) The types of circRNAs. (**C**) Types of differentially regulated circRNAs detected by whole transcriptome sequencing (fold change ≥ 2.0, *p* < 0.05 and FDR < 0.05). The circRNAs were classified into 5 types according to the relationship and genomic loci with their associated coding genes.

circRNAs were widely distributed in all chromosomes, including sex chromosomes X and Y (Figure 2A). The circRNAs were classified into six categories, according to their relation with protein-coding genes: 1.1% were antisense, 4.57% were exonic, 4.01% were intergenic, 1.79% were intronic, and 88.54% were sense overlapping (Figure 2B). Sequencing detected 170 circRNAs that were differentially expressed (fold change ≥ 2, *p* < 0.05 and FDR < 0.05) between PTB patients and healthy individuals, comprising 6 exonic, 8 intergenic, 3 antisense intronic, and 153 sense-overlapping circRNAs. Among these, 102 circRNAs were significantly upregulated and 68 were significantly downregulated in the PTB group compared with the control group (Figure 2C). According to the fold-changes of circRNAs expression between the two groups, the top 20 upregulated and downregulated circRNAs were screened and presented in Table 1.

**Table 1:** Top 20 up-regulated and down-regulated circRNAs in PTB compared with health individuals

Up-regulated circRNAs			Down-regulated circRNAs		
circRNA	FC(abs)	FDR	circRNA	FC(abs)	FDR
circRNA_00074	12.28579975	0.004175	circRNA_01953	0.170315857	0.02636
circRNA_09585	11.63401215	0.007433	circRNA_10381	0.151824411	0.012064
circRNA_01387	10.86328581	0.004742	circRNA_01471	0.14398754	0.006978
circRNA_06439	10.83156631	0.016142	circRNA_13135	0.14397109	0.017432
circRNA_14623	10.62873673	0.020947	circRNA_08841	0.142971033	0.042864
circRNA_05084	10.04303572	0.01169	circRNA_01363	0.141548496	0.042176
circRNA_01149	9.844276082	0.018496	circRNA_08734	0.14103484	0.012314
circRNA_10365	9.476776222	0.026262	circRNA_12825	0.13482315	0.032586
circRNA_02844	9.285017558	0.047858	circRNA_10794	0.121728322	0.048532
circRNA_06231	9.204237431	0.04892	circRNA_05688	0.116294282	0.012948
circRNA_02522	8.773692123	0.036389	circRNA_03345	0.113093168	0.009833
circRNA_08576	8.695696261	0.022961	circRNA_01053	0.112571054	0.034085
circRNA_02523	8.524890891	0.021688	circRNA_07484	0.112124904	0.03517
circRNA_11866	8.490662062	0.044431	circRNA_02590	0.109923013	0.026445
circRNA_07851	7.515927683	0.029571	circRNA_05538	0.106029315	0.024423
circRNA_12109	7.392819406	0.017543	circRNA_13478	0.100383739	0.017499
circRNA_08325	7.168530095	4.92E-05	circRNA_09064	0.092116349	0.03708
circRNA_11528	6.925267904	0.018382	circRNA_06992	0.065623496	0.009276
circRNA_07003	6.860691114	0.031675	circRNA_04993	0.062712573	7.29E-05
circRNA_12685	6.339519969	0.007023	circRNA_09993	0.06180201	0.000857

### Real-time PCR validation of differentially expressed circRNAs

**Figure 3:**
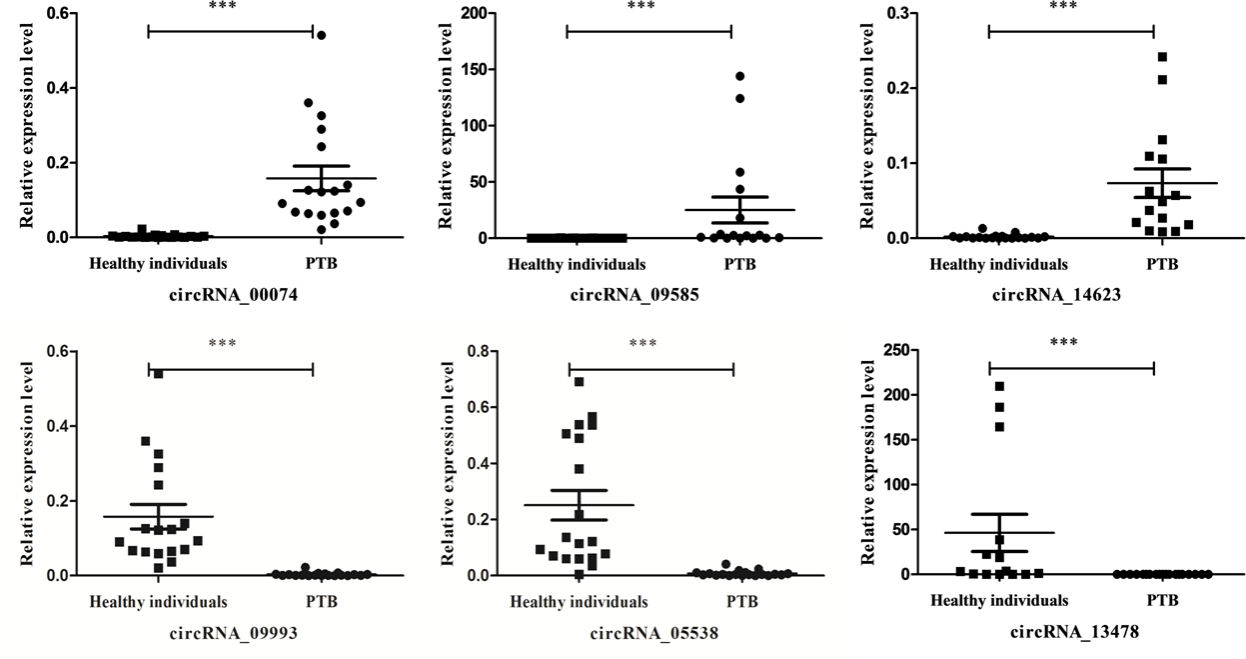
Validation of circRNAs expression with real-time PCR We analyzed the expression of six circRNAs (circRNA_14623, circRNA_09585, circRNA_00074, circRNA_05538, circRNA_09993 and circRNA_13478) from the whole transcriptome sequencing with real-time PCR. The expression level of six circRNAs was measured in 20 PTB patients and 20 healthy individuals. The 2^−ΔΔCt^ was used to normalize the relative gene expression data. Statistical analysis was performed using one way analysis of variance (ANOVA) test. *p* < 0.05 was considered as statistically significant.

To verify the sequencing data, expression of a set of circRNAs (hsa_circRNA_14623, hsa_circ_09585, hsa_circ_005538, hsa_circ_09993, hsa_circ_13478 and hsa_circ_00074) was investigated using real-time PCR (Figure 3). The results were consistent with the sequencing data from the PTB patients, indicating that the sequencing findings were accurate and reproducible.

### Functional annotation of differentially expressed circRNAs

**Figure 4:**
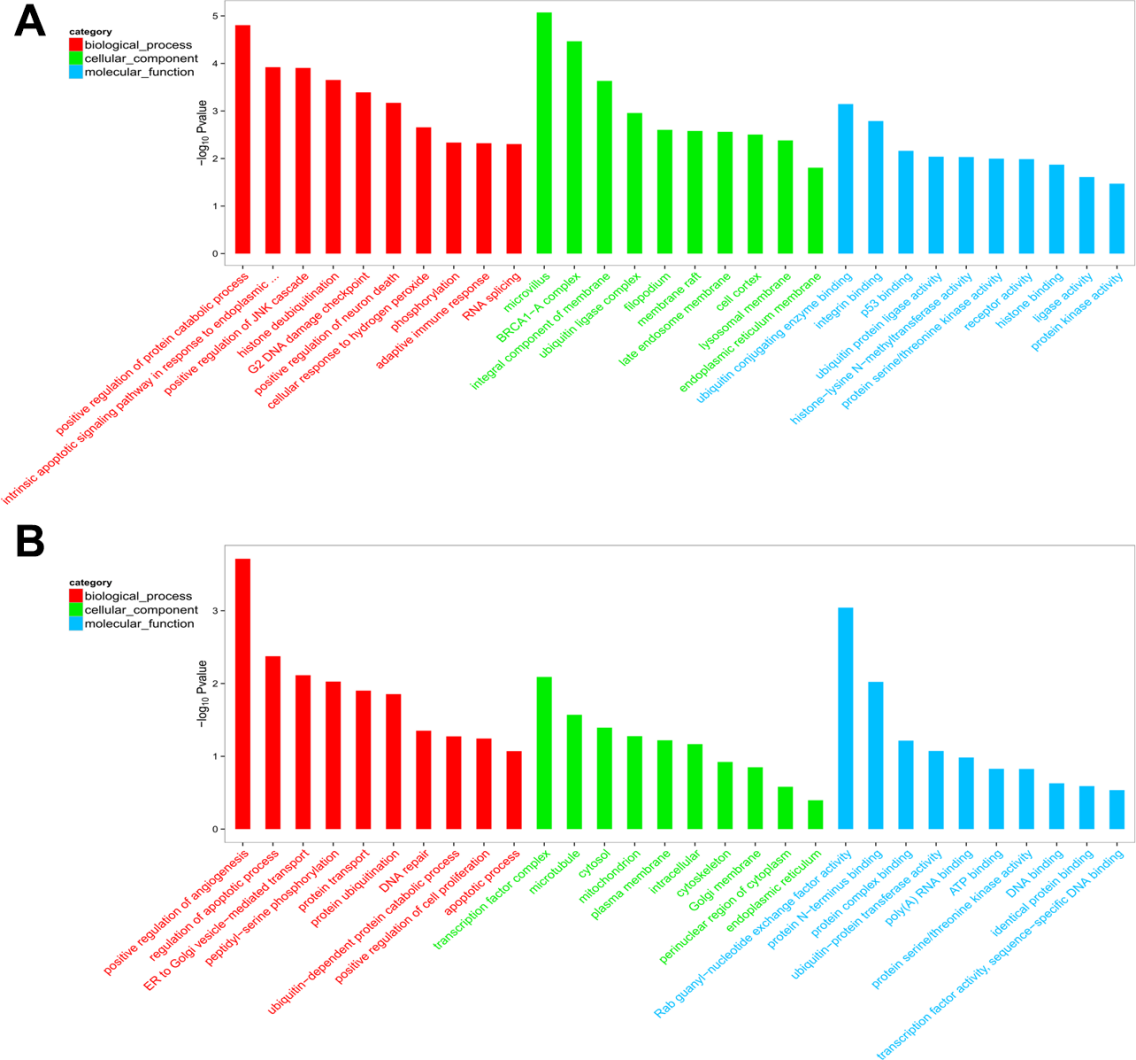
Top 20 GO terms for circRNA co-expression genes between PTB patients and healthy individuals (**A**) GO of upregulated circRNA in PTB patients. (**B**) GO of downregulated circRNAs in PTB patients. The top enriched GO terms in the PTB and healthy individuals were presented by enrichment score. The -log10 (*p*-value) yields an enrichment score representing the significance of GO term enrichment among differentially expressed circRNAs.

**Figure 5:**
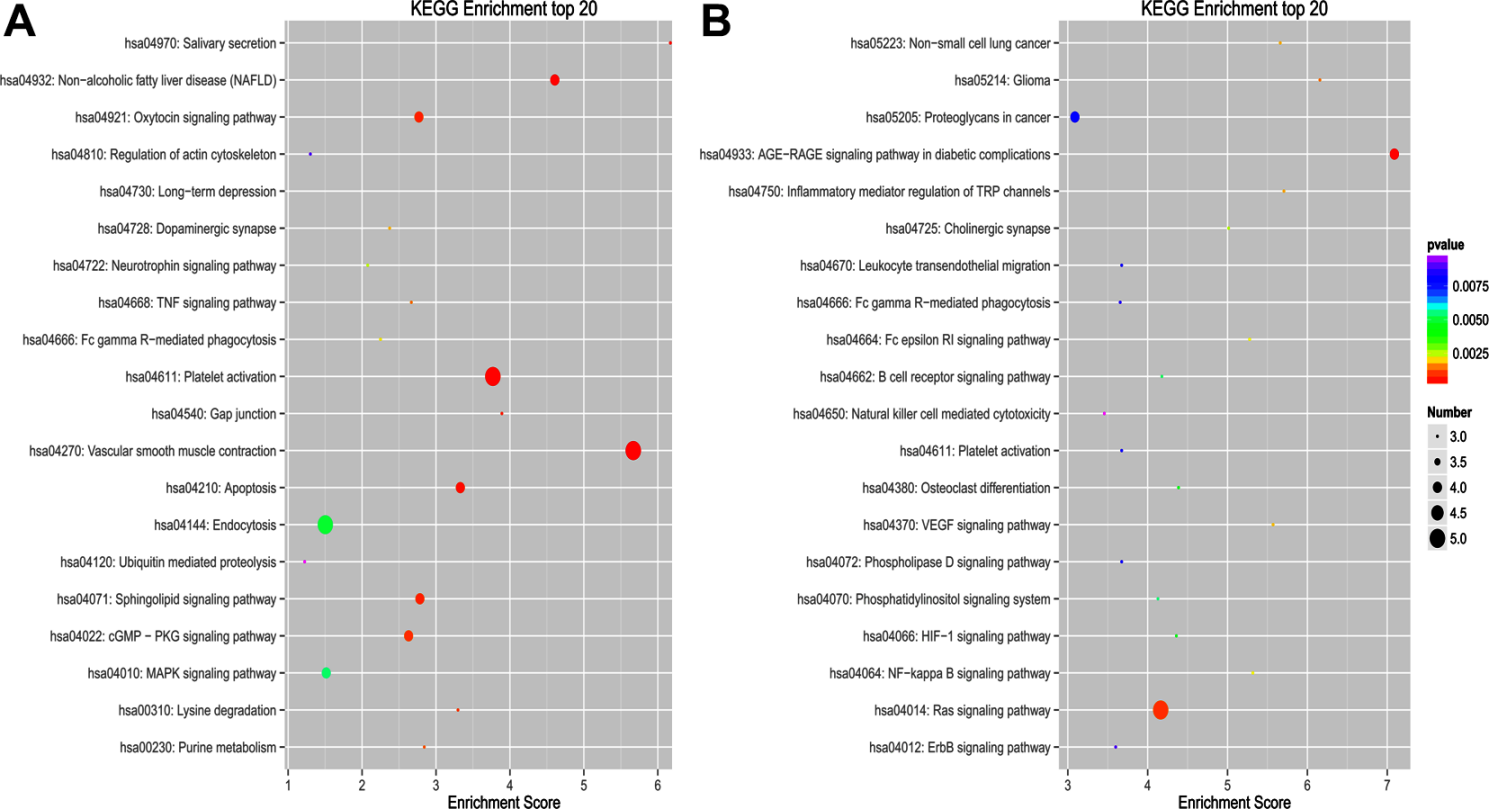
Top 20 KEGG pathways for circRNA co-expression genes between PTB patients and healthy individuals (**A**) KEGG of upregulated circRNA in PTB patients. (**B**) KEGG of downregulated circRNAs in PTB patients. KEGG pathway analysis was performed to determine the involvement of linear transcripts in different biological pathways. The -log10 (*p*-value) yields an enrichment score indicating the significance of pathway correlations.

To explore the putative function of differentially expressed circRNAs, under the assumption that circRNA function is related to the known function of the host linear transcripts, GO (Gene Ontology) annotation and KEGG (Kyoto Encyclopedia of Genes and Genomes) pathway analysis were used to determine the functions of differentially expressed circRNAs between PTB patients and healthy individuals. The top 20 dysregulated GO processes of each subgroup [biological process (BP), cell components (CC) and molecular function (MF)] were analyzed according to the enriched, dysregulated circRNAs derived from the gene annotation. Prediction terms with a *p* value < 0.05 were selected and ranked by their *p* value. According to the routine GO classification algorithms, an enrichment score was used to enrich the significant GO terms of the differentially expressed genes. For the upregulated circRNAs, the top three GO processes included positive regulation of protein catabolic processes, intrinsic apoptotic signaling pathway in response to endoplasmic reticulum stress, and positive regulation of the c-Jun N-terminal kinase (JNK) cascade in the BP subgroup. The microvillus, BRCA1-A complex, and integral component of the membrane were the top three processes in the CC subgroup. Ubiquitin-conjugating enzyme binding, integrin binding, and p53 binding were the top three processes in the MF subgroup (Figure 4A, Supplementary Table 1). The top three GO processes predicted by the downregulated circRNAs were the same as those predicted for the upregulated circRNAs. Enrichment scores were also used to identify the significant GO terms of the differentially expressed circRNAs, in which the top three processes were positive regulation of angiogenesis, regulation of apoptotic process, and endoplasmic reticulum to Golgi vesicle-mediated transport in the BP subgroup; transcription factor complex, microtubules and cytosol in the CC subgroup; and Rab guanyl-nucleotide exchange factor activity, protein N-terminus binding, and protein complex binding in the MF subgroup (Figure 4B, Supplementary Table 2). Furthermore, 112 and 134 KEGG pathways (*p* < 0.05) were identified among the upregulated and downregulated circRNAs, respectively. The top 20 KEGG pathways in the dysregulated circRNAs (upregulated and downregulated circRNAs) are shown in Figure 5A and 5B, and they included the endocytosis pathway (path:hsa04144), pathways in cancer (path:hsa05200), MAPK signaling pathway (path:hsa04010), HTLV-1 infection (path:hsa05166) and ubiquitin-mediated proteolysis (path:hsa04120) signaling pathway (Supplementary Table 3). The result suggests that these pathways are associated with the pathogenesis of PTB.

### Construction of the circRNA–miRNA co-expression network

**Figure 6:**
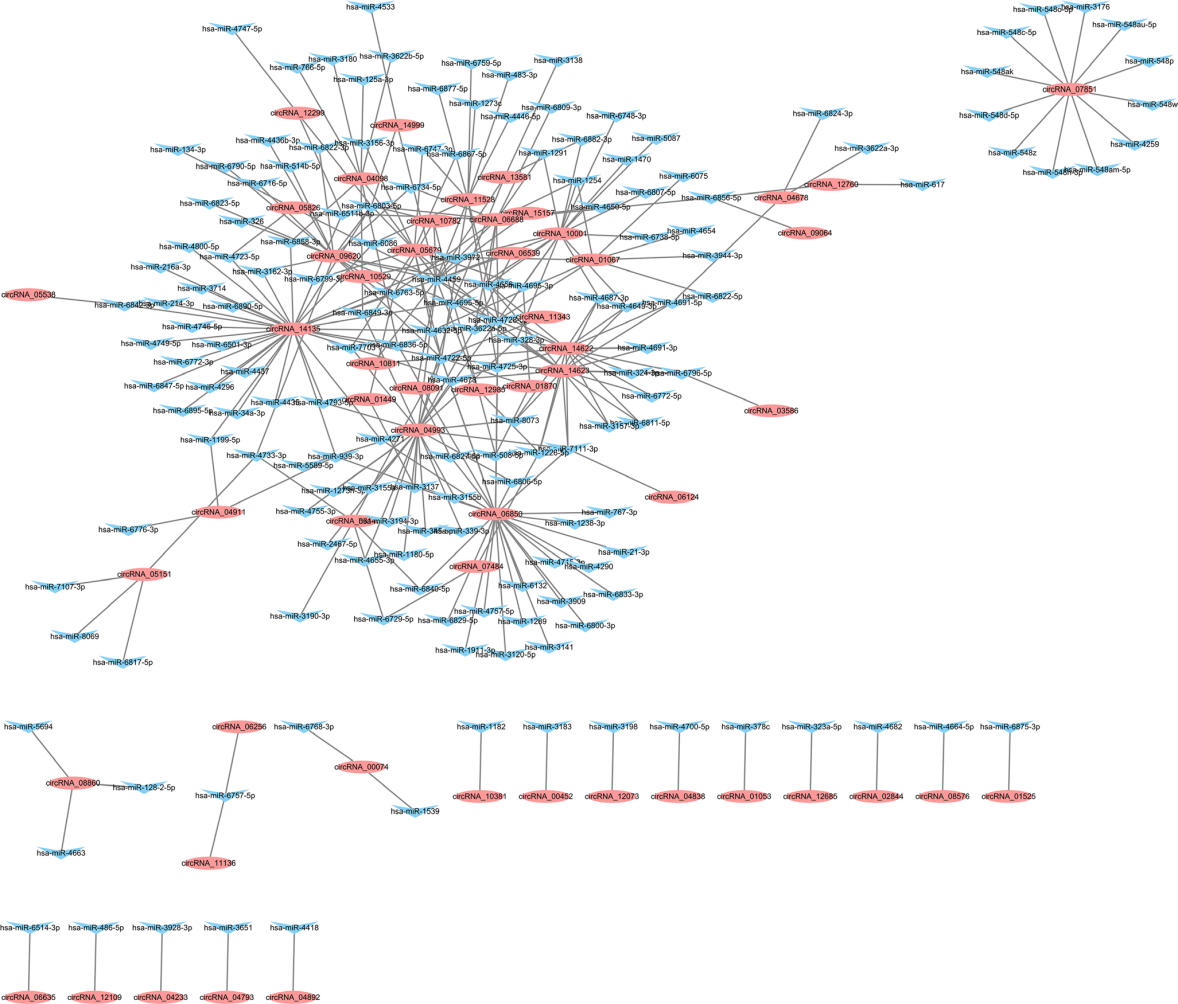
circRNA–miRNA co-expression network Elliptic circular nodes represent circRNAs and triangular nodes represent miRNAs.

In an attempt to reveal the co-expression pattern of circRNA–miRNA, we constructed networks based on the differentially expressed circRNAs. A constructed network map contained the top 300 relationships between circRNAs and miRNAs ranked by *p* value of the hypergeometric distribution. In the network of circRNA–miRNA co-expression in PTB patients compared with the control group, 167 miRNAs interacting with 55 circRNAs among the top 300 relationships were predicted to have closer connections with PTB (Figure 6). For example, members of the miRNA-34 family were direct target genes of p53 and their upregulation induced apoptosis and cell-cycle arrest. The circRNA–miRNA interaction network showed that a single miRNA could be targeted by various circRNAs, whereas a single circRNA could also target different miRNAs. For example, 19 circRNAs were predicted to target hsa-miR-4459, 12 circRNAs to target hsa-miR-4722-5p, and 9 circRNAs to bind hsa-miR-4695-5p in PTB. The co-expression network suggests that miRNA mediates regulation between circRNAs and mRNAs implicated in PTB.

### Construction of ceRNA network

**Figure 7:**
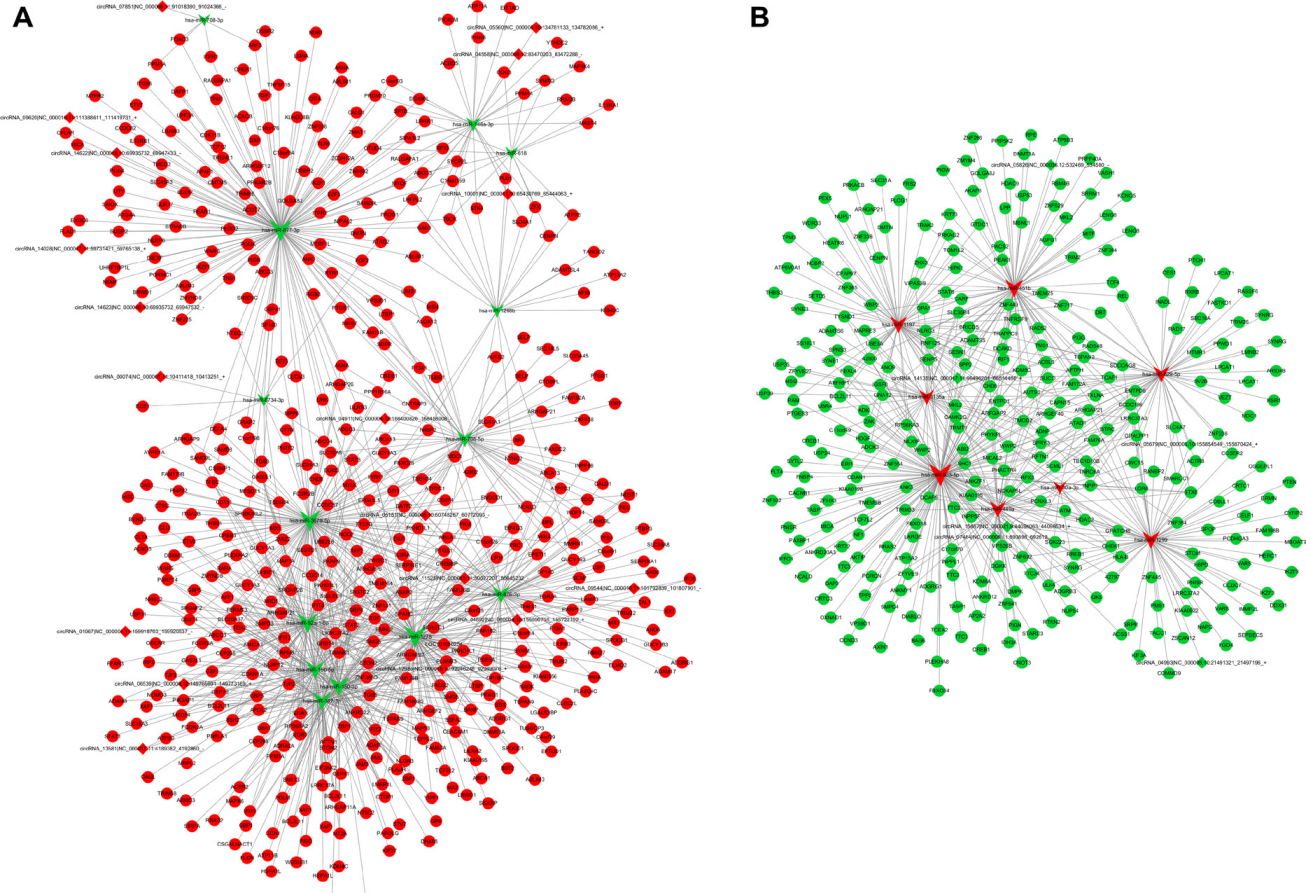
ceRNA network in PTB patients The ceRNA networks were based on circRNA–miRNA–mRNA interactions. The edges represent sequence matching, and circRNAs regulated the expression level of mRNAs via miRNAs. (**A**) ceRNA network contained miRNA downregulation and both circRNA and mRNA upregulation. (**B**) ceRNA network contained miRNA upregulation and both circRNA and mRNA downregulation. Circular nodes, square nodes and triangular nodes represent mRNA, circRNAs, and miRNAs, respectively. Red color represents the upregulated expression, Green color represents the downregulated expression.

Recent studies have shown that RNAs regulate each other with microRNA (miRNA) response elements (MREs) through a mechanism named the ceRNA hypothesis. We constructed a circRNA-associated ceRNA network by integrating the expression profiles and regulatory relationships of the circRNAs, miRNAs and mRNAs from the sequencing data of our 6 samples. A total of 4350 ceRNA relationships was found from the interaction of 41 differentially expressed miRNAs, 2565 differentially expressed mRNAs and 170 differentially expressed circRNAs. According to the expression tendency of the differentially expressed genes in the ceRNAs, two networks were obtained. The first network of 1721 circRNA–miRNA–mRNAs contained 18 upregulated circRNAs and 472 mRNAs and 14 downregulated miRNAs (Figure 7A). The second network of 666 circRNA–miRNA–mRNAs contained both 6 downregulated circRNAs and 307 mRNAs and 8 upregulated miRNAs (Figure 7B). These ceRNA regulatory networks included circRNAs, miRNAs and mRNAs that might play a pivotal role in the pathogenesis of PTB.

## DISCUSSION

Recently, the phenomenon of circRNA has gone from being perceived as a rare curiosity to having a central regulatory role in RNA metabolism [18, 19]. The ever-increasing discovery of circRNA with functional capacity shows that circRNA is a transcriptional product in various tissues and cell types of humans [20], mice [21], *Drosophila* [22], archaea [23] wild boar [24] and rice [25]. To understand the molecular regulatory mechanisms of PTB, we firstly explored the expression profiles of circRNAs in 3 PTB patients and 3 healthy individuals using whole transcriptome sequencing. A total of 170 differentially expressed circRNAs were identified from the patients and healthy individuals; 102 were upregulated and 68 were downregulated. In the expression patterns, 26 circRNAs were upregulated (> 5-fold change), which suggests a role for these differentially expressed circRNAs in the pathogenesis and progression of PTB. In particular, hsa_ circRNA_14623, hsa_circ_09585, hsa_circ_005538, hsa_circ_09993, hsa_circ_00074 and hsa_circ_13478 were confirmed as significantly dysregulated in PTB. These dysregulated circRNAs could be as novel non-invasive biomarker for the diagnosis of PTB that need to be further studied in large patient cohorts.In this study, GO analysis and KEGG pathway annotation were conducted to determine the functions of differentially expressed circRNAs between PTB and healthy individuals. GO enrichment data revealed the target genes that are mainly involved in the regulation of biological processes, cellular components, and molecular functions. The most significant GO items were positive regulation of protein catabolism, apoptotic signaling pathway, endoplasmic reticulum stress, positive regulation of JNK cascade, ubiquitin-conjugating enzyme binding, and integrin and p53 binding, indicating which coding genes contribute to development of PTB. In KEGG pathway analysis, several important pathways closely associated with cancer were identified. These pathways could play pivotal roles in pathogenesis of PTB, including endocytosis pathways in cancer, MAPK signaling pathway, HTLV-1 infection, and ubiquitin-mediated proteolysis signaling pathway. Alveolar macrophages eliminate exogenous foreign substances by endocytic ingestion and subsequent exposure to reactive oxygen species and digestive enzymes. *M. tuberculosis* can escape from the clearance process by inhibiting this fusion of the phagosomes with the lysosomes [26, 27]. The MAPK pathway plays an important role in enhancing antimycobacterial activity and production of immune effector molecules (tumor necrosis factor-α) for defense against mycobacterial infection including phagocytosis, intracellular killing, stimulating T cell activation, and granuloma formation [28]. Accumulating evidence suggests that circRNAs function as miRNA sponges that naturally sequester and competitively suppress miRNA activity to regulate target gene expression [29]. Increasing evidence indicates that aberrant expression of circRNAs promotes cancer pathogenesis by adsorbing cancer-associated miRNAs [30, 31]. Some synthetic circRNAs have exhibited marked anticancer effects, indicating that circRNAs have diagnostic and therapeutic potential [30, 32, 33]. circRNA CiRS-7 contains many binding sites for miR-7, which enables it to absorb this miRNA [34]. A circRNA called sex-determining region Y contains many miRNA-binding sites, and it is reported to function as an miR-138 sponge [35]. Chondrocyte extracellular-matrix-related circRNA regulates matrix metalloproteinase 13 expression by functioning as an miR-136 sponge, and it participates in chondrocyte extracellular matrix degradation [36]. circ-FOXO3 could function as a sponge to bind several potential targeting miRNAs, resulting in increased gene expression and inhibition of tumor growth and angiogenesis [37]. To better understand the regulatory mechanism of circRNA in the pathogenesis of PTB, ceRNAs were constructed and analyzed. ceRNAs have an important influence on regulating gene expression at the post-transcriptional level and are involved in oncogenesis and cancer progression [38]. circRNAs are proposed to harbor miRNAs, and are enriched with functional miRNA-binding sites [25]. To date, there has been no report on ceRNAs in PTB. Here, for the first time, we constructed ceRNA networks with the differential expression of circRNA, miRNA and mRNA from our sequencing data. Two networks were identified according to the differential expression of the ceRNA genes. The first network of 1721 circRNA–miRNA–mRNAs contained 18 upregulated circRNAs and 472 mRNAs and 14 downregulated miRNAs. The second network of 666 circRNA–miRNA–mRNAs contained 6 downregulated circRNAs and 307 mRNAs and 8 upregulated miRNAs. These data suggest that circRNAs harbor MREs and play pivotal regulatory roles in PTB. These results might enrich our understanding of the pathogenesis of PTB.

In summary, we performed whole transcriptome sequencing combined with our small RNA sequencing to investigate potential circRNA-mediated ceRNA interplay by using sample-matched miRNA, circRNA and mRNA expression profiles in PTB patients and healthy individuals in combination with miRNA regulatory network based on “ceRNA hypothesis”. Two PTB-specific dysregulated circRNA-mediated ceRNA networks were constructed, which for the first time enables an overall view and analysis of circRNA-associated ceRNA mediated gene regulation in the pathogenesis of PTB. This study will not only help to understand the circRNA-mediated ceRNA regulatory mechanisms in the pathogenesis of PTB, but also provide useful information for exploring the potential roles of circRNA in PTB.

## MATERIALS AND METHODS

### Ethics statement

This study was approved by the Ethics Committee of the Faculty of Medicine. Written informed consent was obtained from participants prior to their enrollment in the study.

### Patients and healthy individuals

PTB patients were selected according to the following criteria: diagnosis based on clinical manifestations, bacterial culture, and radiographic findings; no major complications, such as chronic obstructive pulmonary disease, asthma, lung cancer, pneumonia, diabetes, and hypertension; no family history of hereditary diseases; no history of HIV infection, cancer, long-term hormone use, and organ transplantation; and no anti-TB medication. There was no family history of hereditary diseases and low immune function in the healthy individuals. There were no significant differences in age and gender between the patients and healthy individuals. All of the patients were secondary tuberculosis. 3 paired samples were selected for whole transcriptome sequencing and 20 paired samples were used for extra evaluation by real-time PCR. Demographic and clinical characteristics of the study population are summarized in Supplementary Table 4. The PTB patients and healthy individuals were recruited from the First People’s Hospital of Zhangjiagang, Jiangsu Province, China, between May and August 2017.

### Blood sampling and RNA extraction

Early morning, fasting whole blood samples were collected from the PTB patients and healthy individuals in 3.0-ml tubes with heparin lithium anticoagulant. Within 4 h of collection, the leukocytes were isolated from the whole blood using an Red Blood Cell Lysis Buffer kit (RT-122-02, Tiangen, Beijing, China). The blood cells were transferred into microcentrifuge tubes with TRIzol reagent (Invitrogen, Carlsbad, CA, USA). Total RNA was quantified with a NanoDrop ND-2000 spectrophotometer (Thermo Fisher Scientific, Scotts Valley, CA, USA), and RNA integrity was assessed using an Agilent Bioanalyzer 2100 (Agilent Technologies, Santa Clara, CA, USA).

### Whole transcriptome sequencing

Whole transcriptome sequencing was quantitatively analyzed by Shanghai OE Biotech. After removal of rRNA and then constructing the library, whole transcriptome sequencing was performed. The clean reads were aligned to the reference genome by Bowtie2 (http://bowtie-bio.sourceforge.net/bowtie2/manual. shtml). For unmapped reads, the junctions were picked out using a back-splice algorithm. Finally, circRNAs were verified with software developed by Shanghai OE Biotech which were considered as the reference sequence for further analysis. Expression of circRNA was measured by RPM.

### Annotation of host linear transcripts and identification of differentially expressed circRNAs

There were two selection criteria for differentially expressed circRNAs: ≥ 2-fold change in the same circRNA in PTB patients and healthy individuals; and *p* < 0.05 and false discovery rate (FDR) < 0.05, which was used to control filtration upon the statistics of alignment quality scores. Each circRNA was calculated with *p*-value (*p* < 0.05). The false discovery rate (FDR) was used to evaluate the significance of the *p*-value. DESeq is an R package to analyze count data from whole transcriptome sequencing data and test for differential expression (http://bioconductor.org/packages/release/bioc/html/DESeq.html). Linear transcripts were annotated according to the location of the chromosome where the circRNA sequence was overlapped. We compared the circRNAs with genetic elements to explore the distribution of identified circRNAs in the genome.

### GO annotation and KEGG pathway analysis of linear transcripts

DAVID (Database for Annotation, Visualization and Integrated Discovery) was used to analyze the potential functions of linear transcripts. Gene functions were classified into three subgroups: BP, CC and MF. The top enriched GO terms in the PTB and healthy individuals were presented by enrichment score. The -log10 (*p*-value) yields an enrichment score representing the significance of GO term enrichment among differentially expressed circRNAs. KEGG pathway analysis was performed to determine the involvement of linear transcripts in different biological pathways. The -log10 (*p*-value) yields an enrichment score indicating the significance of pathway correlations.

### miRNA target prediction

Evidence shows that circRNAs bind with miRNAs and function as natural miRNA sponges to influence related miRNA activities. Interactions between miRNAs and circRNAs were evaluated using miRanda. A hit between any expressed miRNA (including the new predicted miRNA) and a target circRNA was considered for a miRanda score ≥ 140, corresponding to at least a perfect seed match.

### circRNA–miRNA co-expression network analysis

A circRNA–miRNA co-expression network was based on the prediction of miRNA binding sites and the correlations between circRNA and miRNA that was ranked by miRanda according to *p* value of the hypergeometric distribution. The top 300 circRNA–miRNAs with lowest *p* value were selected to generate a network map with Cytoscape software version 3.2.1. Elliptic circular nodes represented circRNAs and triangular nodes represented miRNAs.

### ceRNA network analysis

The circRNAs, miRNAs and mRNAs with expression levels that shared a meaningful correlation were subjected to ceRNA analysis. Potential MREs were searched for in the circRNAs and mRNA sequences, and overlapping of the same miRNA seed sequence binding sites in both the circRNA and mRNA sequences was considered to predict circRNA–miRNA–mRNA interaction. miRNA binding sites were predicted with miRcode (http://www.mircode.org/), and miRNA–mRNA interactions were predicted with TargetScan (http://www.targetscan.org/).

### Real-time PCR

To validate the data from RNA sequencing, 6 circRNAs were selected and validated with real-time PCR. Specific divergent primers for each circRNA were designed according to the sequence of the linear transcripts, and all divergent primers are shown in Supplementary Table 5. Total RNA was extracted from the blood, digested using RNase R and purified. cDNA was synthesized using the EasyScript One-Step gDNA Removal and cDNA Synthesis SuperMix (Transgen Biotech, Beijing, China). Real-time PCR was performed on a CFX96 Touch Deep Well Real-Time PCR Detection System (Bio-Rad, , USA) according to the manufacturer’s instructions for the iTaq Universal SYBR Green Supermix (Bio-Rad, USA). The real-time PCR program was initiated by denaturation at 95°C for 1 min, followed by 40 cycles of 95°C for 5 s and 60°C for 30 s. Expression of circRNAs was normalized to β-actin (internal standard control) and calculated using the 2^−ΔΔCt^ method. All experiments were done in triplicate.

### Statistical analysis

GraphPad Prism for Windows version 5.0 (GraphPad Software, San Diego, CA, USA) was used to plot all graphs. Statistical analysis was performed using one way analysis of variance (ANOVA) test. *p* < 0.05 was considered as statistically significant.

## SUPPLEMENTARY MATERIALS TABLES








