# Supplementary material for: Identification and comparison of novel circular RNAs with associated co-expression and competing endogenous RNA networks in pulmonary tuberculosis
Source: Oncotarget. 2017 Nov 27;8(69):113571–82. doi: 10.18632/oncotarget.22710 (PMC5768347; doi:10.18632/oncotarget.22710)
Supplement: Supplementary file 1 [file oncotarget-08-113571-s001.pdf]

Identification and comparison of novel circular RNAs with associated co-expression and competing endogenous RNA networks in pulmonary tuberculosis

SUPPLEMENTARY MATERIALS

Supplementary Table 1: GO enrichment of upregualted circRNAs. See Supplementary_Table_1

Supplementary Table 2: GO enrichment of downregualted circRNAs. See Supplementary_Table_2

Supplementary Table 3: KEGG enrichment. See Supplementary_Table_3

Supplementary Table 4: Characteristic of study participants with PTB patients and healthy individuals

Characteristic	Pulmonary tuberculosis (TB)	Healthy Controls
Total Number	20	20
Gender (male/female)	20 (15/5)	20 (11/9)
Age (years;average;range)	46.2 (20–72)	45 (21–69)
BCG vaccination (Yes/NO)	14/6	20/0
Smear test (Positive/Negative)	Positive	NA
History of smoking (Yes/No)	7/13	NA
Sputum culture-proven	Positive	NA
Chest X-ray and CT-proven	15	NA

NA: Not applicable, BCG: Bacille Calmette-Guerin vaccine.

Supplementary Table 5: Primers used for real-time PCR

Primers	Sequence (5′ to3′)	nt
circRNA_00074-1	ATACTCTATCCCGTTGTGCAC	21
circRNA_00074-2	AGAAGGGCACAGGGAAGTG	19
circRNA_09585-1	CACTTGACGTCAGGAGATCACA	22
circRNA_09585-2	GTGAACCGAAGTTTAGCAATGG	22
circRNA_14623-1	CAATGCAGTTTCCTTCCTCTC	21
circRNA_14623-2	GGCTGAATTGATAGAGAATGG	21
circRNA_05538-1	TTCTGCTGGTGTCTGCTGCTG	21
circRNA_05538-2	TGTATGAGGATGGAGAAGACC	21
circRNA_09993-1	AATTCACTGCGTTGTCGGAC	20
circRNA_09993-2	CCTCTTTGATGATAAAGCAGTG	22
circRNA_13478-1	TTTGGAGTTCTTCCCTGGG	19
circRNA_13478-2	GATGAGCTCAGCTTTGAACAG	21
β-actin-1	TCATGAAGTGTGACGTTGACATCCGT	26
β-actin-2	CCTAGAAGCATTTGCGGTGCACGATG	26
